# Deep Encrypted Traffic Detection: An Anomaly Detection Framework for Encryption Traffic Based on Parallel Automatic Feature Extraction

**DOI:** 10.1155/2023/3316642

**Published:** 2023-03-10

**Authors:** Gang Long, Zhaoxin Zhang

**Affiliations:** Faculty of Computing, Harbin Institute of Technology, Harbin 150000, China

## Abstract

With an increasing number of network attacks using encrypted communication, the anomaly detection of encryption traffic is of great importance to ensure reliable network operation. However, the existing feature extraction methods for encrypted traffic anomaly detection have difficulties in extracting features, resulting in their low efficiency. In this paper, we propose a framework of encrypted traffic anomaly detection based on parallel automatic feature extraction, called deep encrypted traffic detection (DETD). The proposed DETD uses a parallel small-scale multilayer stack autoencoder to extract local traffic features from encrypted traffic and then adopts an L1 regularization-based feature selection algorithm to select the most representative feature set for the final encrypted traffic anomaly detection task. The experimental results show that DETD has promising robustness in feature extraction, i.e., the feature extraction efficiency of DETD is 66% higher than that of the conventional stacked autoencoder, and the anomaly detection performance is as high as 99.998%, and thus DETD outperforms the deep full-range framework and other neural network anomaly detection algorithms.

## 1. Introduction

With the increasing scale of Internet users, the Internet has begun to carry an increasing number of emerging network applications, and accurate traffic classification is the premise of the basic tasks of the network. Especially with the wide application of encryption data transmission, network traffic encryption is becoming a standard [1–4]. Encryption will make abnormal behaviors in the network such as botnet [5], worm [6], image transmission [7, 8], and denial of service attack [9] more covert. Therefore, how to detect malicious encryption traffic without decryption is the present difficulty of traffic monitoring, which poses new challenges to traffic anomaly detection.

As a heuristic work, Anderson and Mcgrew proposed expanding the existing traffic anomaly detection method without decrypting the network traffic [10–12]. In their work, the feature set with prominent discrimination is selected from unencrypted transport layer security (TLS) handshake information, DNS response information related to the destination IP address in TLS flow, and header information of HTTP flow within the 5-minute window of the same IP source address, and the network traffic with malicious behavior is identified from encrypted network traffic by the machine learning method. Inspired by efficient feature extraction capabilities of deep learning technology [13–15], Wei et al. [16] used a one-dimensional convolutional neural network (1D-CNN) to better fit encryption traffic data based on Anderson and other predecessors' work. In 2018, Yang et al. [17] proposed two deep learning methods to classify encryption traffic. One is to extract encrypted traffic features from the autoencoder, and the other is to use a convolutional neural network to learn high-dimensional features of encrypted traffic. Both deep learning methods can extract features from stream metadata, package size, package arrival time, and unencrypted TLS header information. Moreover, their experimental results verified that the convolutional neural network is superior to the autoencoder, as well as other competitive algorithms, for feature extraction. Zeng et al. proposed a deep full-range (DFR) anomaly detection framework [18]. Generally speaking, for traditional machine learning anomaly detection algorithms, a machine learning network (such as LSTM and SAE) was first used to extract features. After feature extraction, the L1 regularization method was used to screen features to reduce the computation of anomaly detection.

However, the traditional machine learning methods using statistics for traffic anomaly detection have great disadvantages in feature selection. First of all, the quality of feature selection depends not only on strong expert information but also on private information to a certain extent, which is undoubtedly a very resource-consuming task. Moreover, deep learning methods for traffic anomaly detection, such as Wei's method [16], focused only on the structure of the deep neural network model to extract many payload bytes from the original traffic data packages. In addition, all the extracted payload bytes are global features in traffic data packages, which may have the fatal weaknesses, such as feature dimension redundancy, a large computation amount, poor detection performance, and insufficient feature extraction efficiency.

To overcome the shortcomings of the above methods, we propose an encrypted traffic anomaly detection framework based on parallel automatic feature extraction, called deep encryption traffic detection (DETD), in this work. The proposed DETD extracts local features in encrypted traffic by using small-scale parallel stacked self-encoder layers and then uses feature filtering to extract effective information that strongly indicates encrypted traffic. Specifically, the parallel feature extraction module used in DETD effectively retains the characteristics of encrypted traffic, which can improve feature extraction efficiency and effectively reduce the delay of encrypted traffic classification. The main contribution of this paper lies in the following three aspects:

1. We propose an encrypted traffic anomaly detection framework, including encrypted traffic packet pretreatment, parallel automatic feature extraction, feature selection, and anomaly detection classification.
2. We introduce a small-scale parallel automatic feature extraction algorithm that can effectively extract the local features of encrypted traffic and greatly improve feature extraction efficiency.
3. We design an L1 regularization-based feature selection algorithm to select the most representative feature set for the final encrypted traffic anomaly detection task.

The rest of this paper is organized as follows.  Section 2 presents the related work.  Section 3 introduces the proposed DETD framework. Sections 4 and 5 present the experimental evaluation and discussion, respectively.  Section 6 concludes the paper.

## 2. Related Work

Although traditional traffic anomaly detection methods have made certain progress [19–22], most traditional anomaly detection algorithms are not suitable for encrypted traffic. In the problem of traffic anomaly detection, encrypted traffic communication and unencrypted traffic communication greatly differ. First of all, the traffic features after encryption have changed greatly, and most content-based anomaly detection methods, such as deep package detection algorithms, are difficult to apply to encrypted traffic algorithms [23]. Second, encryption protocols are often accompanied by traffic masquerading techniques (such as protocol confusion and protocol variation), which transform encrypted traffic characteristics into commonly used traffic characteristics, bringing great difficulties to traffic anomaly detection [24]. Because encryption technology encrypts only the payload, the anomaly detection method based on data stream characteristics is less affected by encryption. According to the different ways in which data stream features are used for encrypted traffic anomaly detection, we can divide these encrypted traffic anomaly detection methods into the following two categories: (1) manual feature selection method and (2) automatic feature extraction approach.

The anomaly detection methods based on manual feature extraction extract feature sets that are helpful for anomaly detection through expert information, such as the duration of the stream, the number of bytes of the stream per unit time, the arrival times in the forward and backward directions, and the size and density distribution of the stream. Lakhina et al. used the distribution of data package characteristics (IP address and port) to detect and identify large-scale anomaly traffic [25]. The experimental results showed that the clustering method can effectively divide normal traffic and anomalous traffic into different clusters and can be used to find new anomalous traffic. Soule et al. proposed a method based on a traffic matrix to identify anomalous traffic [26]. In their method, Kalman filtering was first used to identify normal traffic, and then the threshold, variance analysis, wavelet transform, and generalized likelihood ratio were used to identify anomalous traffic. The ROC curve showed that their method can achieve a better balance between false positives and false negatives.

The core of anomaly detection methods based on automatic feature extraction is to use the powerful fitting ability of deep neural networks to automatically extract feature sets suitable for anomaly detection tasks from encrypted traffic for final anomaly classification. Compared with traditional machine learning methods, automatic feature extraction methods have a deeper level of learning ability. Therefore, these approaches have a wide range of application scenarios in industry or academia, such as machinery fault diagnosis [27–29], network stream detection [30], botnet detection [31], and intrusion collaborative detection [32]. As mentioned in these references, the automatic feature extraction methods always adopt neural networks to extract features. For example, Odiathevar et al. [30] developed an online offline framework for anomaly traffic detection in network streams. In this framework, the authors adopted the learned knowledge of the offline model as the bias for selecting the training data for the online model, so that it can be used with any deep learning method and any anomaly detection algorithm. Moreover, Kim et al. [31] proposed a botnet detection method that can capture periodicity in network data, which is the key to detecting various botnets exhibiting sequential patterns by using recurrent neural networks. The proposed method also can detect botnets in an online manner based a new anomaly scoring function representing the maliciousness of network connections. Similar to this work, Wang et al. [32] introduced an intrusion collaborative detection framework based on confidence.

However, these aforementioned approaches may have certain shortcomings. First, the manual feature selection method requires expensive expert information and labor costs, and thus the advantages and disadvantages of selecting features depend entirely on expert experience. In addition, the automatic feature extraction method is inefficient in feature extraction, which may have a certain redundancy in feature extraction and may have the defects of poor detection performance and a large calculation amount.

## 3. The Proposed DETD Framework

**Figure 1:**
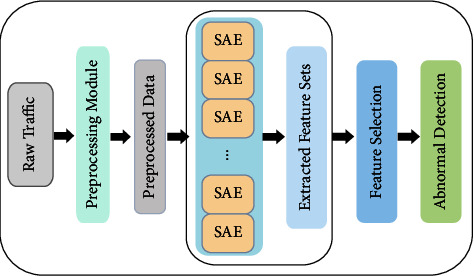
The proposed DETD framework.

In this section, we present our proposed DETD encrypted traffic anomaly detection framework in detail. As shown in Figure 1, the proposed DETD can be divided into four functional modules: the encrypted data package preprocessing module (pretreatment module), parallel SAE automatic feature extraction module (PSAE module), feature selection module, and anomaly detection module. The encryption data package preprocessing module is mainly used to clean the interference data in the data package, repeat files, and read the original traffic data package.

### 3.1. Pretreatment Module

**Figure 2:**
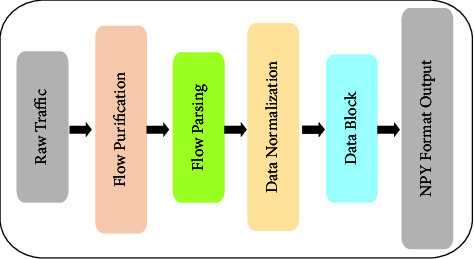
The four steps in the pretreatment module of DETD.

The data package pretreatment module mainly reads the contents of encrypted traffic data packages through data package analysis and then converts them into the data format required by subsequent modules. The main reasons for adopting pretreatment are as follows: (1) the original data contain information that may interfere with anomaly detection, such as port numbers or MAC addresses; and (2) the original traffic data from the network have different scales, which is not an ideal input format for a deep neural network model. The preprocessing module includes four steps: flow purification, flow parsing, data normalization, and data block, as shown in Figure 2.

(i) *Flow Purification*. This step ensures that our proposed method is free from interference data, duplicate files, and empty files in the traffic packets. The data flow purification process is used to screen some of the TCP or UDP headers, some of the data link layers, and Ethernet-related data, such as MAC addresses.
(ii) *Flow Parsing*. In this data stream parsing process, we use Python's third-party standard library, the *Scapy* library, to read data from the encrypted stream packets. Notably, data feature recovery may cause data feature loss. To retain the data feature as much as possible, each byte in the stream data is set as the feature value *x*_*m*_^*n*^, where *x*_*m*_^*n*^ ∈ *D*^*d*^ (d is the dimension of dataset D), and *x*_*m*_^*n*^ represents the feature of column *n* in the *m*-th data stream. Considering that the following feature extraction modules need a unified input format, we fill the analyzed data according to the overall data situation. First, set a maximum investigation value (MIV), which indicates the number of data features contained in each piece of data x_*m*_, and then fill the data with zero padding if the number of data features *n* is less than MIV. Otherwise, the redundant feature data will be truncated. By default, we set MIV as 784 dimensions. Each piece of data is calculated as follows:
  (1) $$\begin{array}{c} x_{m}^{n}=\left\{\begin{array}{c} \left(x_{m}^{1},x_{m}^{2},\ldots ,x_{m}^{i},0_{m}^{i+1},\ldots ,0_{m}^{MIV}\right)\;if\;n<MIV\\ \left(x_{m}^{1},x_{m}^{2},\ldots ,x_{m}^{i}\right)\;if\;n\geq MIV \end{array}\right.\text{.} \end{array}$$
(iii) *Data Normalization*. Different evaluation indices often have different dimensions and dimensional units. The data normalization processing formula eliminates the dimensional influence between indices and improves comparability between data indices. After normalization of the original data, each index is on the same order of magnitude, which is suitable for comprehensive comparative evaluation. The calculation formula is as follows:
  (2) $$\begin{array}{c} {x^{*}}_{m}^{n}=\frac{x_{m}^{n}-\min\left(x_{m}^{j}\right)}{\max\left(x_{m}^{i}\right)-\min\left(x_{m}^{j}\right)}\text{,} \end{array}$$
where max(*x*_*m*_^*i*^) and min(*x*_*m*_^*j*^) are the parts with the largest and smallest feature values in the *m*-th piece of data, respectively.
(iv) *Data Block*. First, dataset *D* is randomly divided into training sets *D*^train^ and *D*^test^ at a ratio of 7 : 3. Then, we divide the obtained training sets and test sets into data blocks. To extract lightweight features, a piece of data *x*_*m*_ is divided into several equal parts on average. Here, we divide a piece of data into 28 equal parts, with an average of 28 bytes per block.

To better illustrate the pretreatment module of DETD, we summarize it as Algorithm 1. In particular, let the original encrypted traffic data G(*g*_1_, *g*_2_,…, *g*_*m*_) be the preprocessed dataset, and let *PT*_*i*_^*n*^ denote the *i*-th data in *RT*^*n*^. After data preprocessing, a pure encrypted traffic dataset can be generated for the next step of DETD.

### 3.2. Parallel SAE Feature Extraction Module

**Figure 3:**
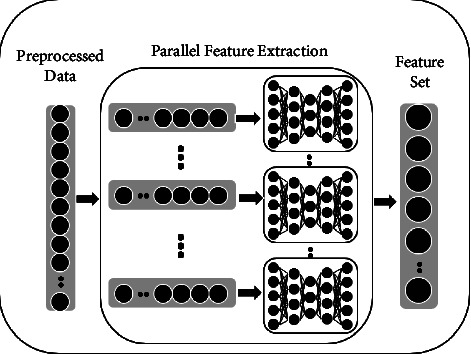
Schematic diagram of PSAE module.

The core of the entire DETD framework is the parallel SAE automatic feature extraction module, called *PSAE*. It is a characteristic of parallel extraction of preprocessed encryption traffic packages by a small-scale stacked autoencoders (SAEs) [33]. The parallel automatic feature extraction module in the DETD framework is shown in Figure 3. The parallel SAE feature extraction module comprises two steps: a parallel SAE training process and a hyperparameter tuning process:

(i) *PSAE Training*. Let *X* be the preprocessed encrypted traffic data packet and *X*′ be the reconstructed data packet from the SAE, where X=[*x*_1_, *x*_2_,…, *x*_*MIV*_] and *X*′=[*x*_1_, *x*_2_,…, *x*_*MIV*_]. After segmenting *X*, we can obtain *m* encrypted traffic data segments *g*_*i*_, where *G*={*g*_1_, *g*_2_,…, *g*_*m*_}. After each small-scale stacked autoencoder is trained, we can obtain the extracted feature set *F*, where *F*={*f*._1_, *f*._2_,…, *f*._*m*_}. Let Q_*SAE*_(X) represent the parallel SAE training process, *h*(*z*) be the sigmoid function, and *J*(*X*) denote the objective function. We have
  (3) $$\begin{array}{c} h\left(z\right)=\frac{1}{1+e^{-z}}\text{,}\\ f_{i}=Q_{SAE}\left(g_{i}\right)\left(1\leq i\leq m\right)\text{,}\\ J\left(X\right)=\left\|X-X^{\prime }\right\|\text{.} \end{array}$$
(ii) *Hyperparametric Tuning*. Because the parameters (such as learning rate, number of iterations, and batch size) for feature extraction differ between traffic data blocks, we need hyperparameter tuning on the parallel SAE feature extraction module to avoid overfitting our model. At the same time, to generate an optimal model, the predefined loss function of a given piece of data is minimized to avoid overfitting. We use the root mean square propagation algorithm (RMS-Prop) to train the model. RMS-Prop is an optimizer with pseudo-curvature information that normalizes the gradient using the size of the nearest gradient. It is also a robust optimizer that handles random targets well, making it suitable for microbatch learning. As an optimizer, it normalizes the gradient using the size of the nearest gradient. In addition, it can handle random targets well, making it suitable for microbatch learning. Finally, we output features extracted by hidden layers (see Algorithm 2 for more details). After this process, we obtain a preliminary set of features for anomaly detection classification.

### 3.3. Feature Selection Module

The method of extracting features by the parallel SAE feature extraction module adopts an unsupervised learning method. Not all of the extracted features are helpful for classification tasks. Moreover, even cases where redundant features lead to poor detection may arise. In the DETD framework, an L1 regularization-based feature selection method is adopted to select the *p*-th feature that contributes to classification as an input for anomaly detection [34]. The L1 regularization-based feature selection method is based on the SVM linear kernel.

Given a dataset *D*={(*x*_1_, *y*_1_), (*x*_2_, *y*_2_),…}, where x_*i*_ ∈ *R*^*d*^, *y* ∈ {−1,1}, *y*_*ω*_(*x*_*i*_) is the classification result of the linear SVM, *y* is the data label, and *α* denotes the weight control coefficient, we have the following L1-based loss function:

(4) $$\begin{array}{c} J\left(\omega \right)={\left[y_{\omega }\left(x_{i}\right)-y\right]}^{2}+\alpha \underset{i}{\sum }\left\|\omega_{i}\right\|\text{.} \end{array}$$

By scaling the value of *α*, the intensity of the L1 word weight attenuation can be controlled. Thus, we can optimize the weight *ω*_*i*_ with a low feature contribution rate to 0 to retain the top *P* features that contribute substantially to anomaly detection classification for the final intrusion detection module.

### 3.4. Anomaly Detection Module

The anomaly detection module is mainly used to train a classifier with superior detection performance on the feature set for final anomaly detection. An integrated learning approach, the *AdaBoost* classifier [35], is used in the DETD framework. Let *D*^train^={(*x*_1_, *y*_1_), (*x*_2_, *y*_2_),…, (*x*_*n*_, *y*_*n*_)} be the training set, where *x*_*i*_ ∈ *X* ∈ *R*^*n*^, *y*_*i*_ ∈ *Y* ∈ {−1,1}. The number of iterations is set to *M*. We can divide the anomaly detection module into the following steps:

(i) Initialize the weight distribution of the training:
W_1_=(*ω*_1,1_, *ω*_1,1_,…, *ω*_1,*n*_), *ω*_1,*i*_=(1/*n*), i=1,2,…, n.
(ii) When m ≤ M:
  (a) Use the weight distribution *W*_*m*_ training set to learn and then obtain a weak classifier *f*_*m*_(*x*).
  (b) Calculate the classification error on the training set *error*_*m*_ as follows:
    (5) $$\begin{array}{c} {error}_{m}=\overset{n}{\underset{i=1}{\sum }}\omega_{m,i}I\left(f_{m}\right.\left(x_{i}\right)\neq \left.\left.y_{i}\right)\right)\text{.} \end{array}$$
  (c) Calculate the weights in the strong classifier:
    (6) $$\begin{array}{c} \alpha_{m}=\frac{1}{2}\log\frac{1-e{rror}_{m}}{{error}_{m}}\text{.} \end{array}$$
  (d) Update the weight distribution of the training set:
    (7) $$\begin{array}{c} \omega_{m+1,i}=\frac{\omega_{m,i}}{z_{m}}\exp\;\left(-\alpha_{m}y_{i}f_{m}\left(x_{i}\right)\right)\text{,} \end{array}$$
  where
    (8) $$\begin{array}{c} z_{m}=\overset{n}{\underset{i=1}{\sum }}\omega_{m,i}\exp\;\left(-\alpha_{m}y_{i}f_{m}\left(x_{i}\right)\right)\text{.} \end{array}$$
(iii) The resulting classifier is
  (9) $$\begin{array}{c} F\left(x\right)=sign\left(\overset{n}{\underset{i=1}{\sum }}\alpha_{m}f_{m}\left(x_{i}\right)\right)\text{.} \end{array}$$

## 4. Experimental Evaluation

This section describes the datasets, evaluation metrics, and experimental results of DETD.

### 4.1. Experimental Dataset and Environment

As introduced by Dainotti et al. [36], the lack of multiple shareable traffic datasets as test data is the most obvious obstacle to traffic classification progress. In our experiment, we used the CTU-13 malicious traffic dataset (provided by the Czech University [37]) to test the performance of DETD. The dataset we selected was 3.71 GB in size, and the format was PCAP. After data preprocessing, 1.16 million pieces of data were obtained, of which 800,000 were used as the training set and 360,000 were used as the test set. The experimental environment is configured as follows: Windows 10 system, CPU i7-7700hq, 16 G memory size, and a 1060 GPU. The software frameworks for machine learning are TensorFlow and Sklearn.

### 4.2. Evaluation Metrics

Three common metrics are used to measure the performance, i.e., accuracy, recall, and *F*1_score. Accuracy is used to describe the number of correct predictions over all predictions. Recall refers to the number of positive cases correctly predicted by the classifier in the data. *F*1_score is used to measure both precision and recall. Mathematically,

(10) $$\begin{array}{c} accuracy=\frac{TP+TN}{TP+FP+FN+TN}\text{,}\\ recall=\frac{TP}{TP+FN}\text{,}\\ F1\_score=2*\frac{precision*\;recall}{precision+recall}\text{,} \end{array}$$

where TP is true positive, namely, the number of correctly classified cases as a specific class; FP is false positive, i.e., the number of misclassified cases that are classified as positive class; FN, false negative, is the number of cases that should be classified as positive, rather than a negative result; TN, true negative, is the number of cases that are correctly classified as not that specific class; and precision shows how many of the positive predictions made are correct (true positives).

### 4.3. Comparison with State-of-the-Art Methods

We use our proposed method and eight state-of-the-art automatic feature extraction algorithms [10–12, 17, 18, 30–32] to detect the anomaly of encryption traffic data.  Table 1 compares the proposed algorithm with these anomaly detection algorithms. For a better comparison, we only list the best-performing accuracy parameters.

According to Table 1, the experimental results of the DETD framework are obviously better than those of the manual feature extraction method, and the manual feature extraction algorithm has many limitations. First, the complex function uses limited samples and calculation units, the computing power is limited, and its generalization ability is limited by complex classification issues. More importantly, shallow models have features that require manual sample extraction. However, manually extracting features is a very laborious task, and the excellent features are largely determined by experience and luck. Using deep learning to extract features is advantageous because it can control the number of hidden layer nodes to a polynomial multiple of the number of input nodes instead of presenting an exponential multiple and has strong expressive power. Compared with these deep learning frameworks (such as [30–32]), DETD has great advantages in feature extraction. We can observe that local features for these deep learning methods are better than global features in the problem of encryption traffic anomaly detection. Moreover, the proposed DETD anomaly detection algorithm has improved the AUC index by almost 2.5 effective point redirects. At the same time, the DETD framework is superior to other deep learning feature extraction frameworks in terms of time and computational cost, and the detection performance has also improved. Specially, the used L1-based feature selection in DETD improved the interpretability of using deep learning algorithms for encrypted traffic anomaly detection (see Discussion for more details).

## 5. Discussion

### 5.1. Why Did We Choose Parallel Automatic Feature Extraction?

**Figure 4:**
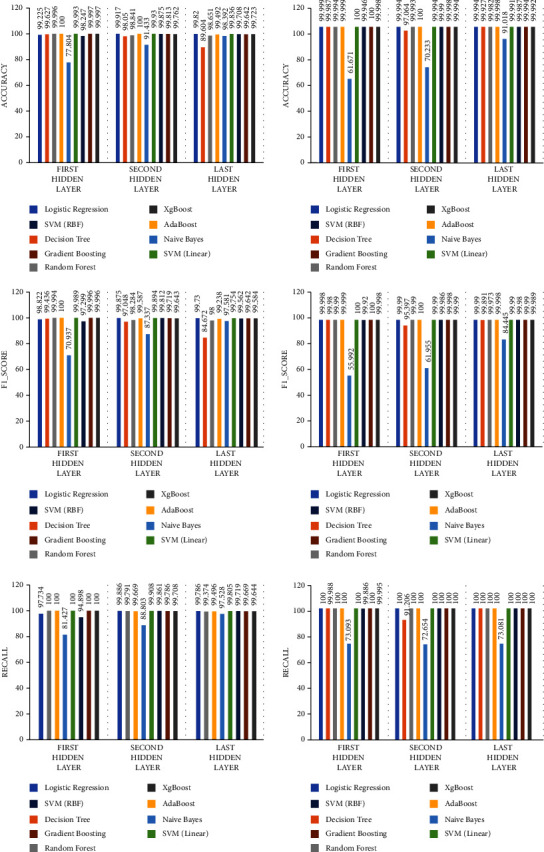
The left column indicates the three experimental evaluation metrics (accuracy, *F*1_score, and recall) of the unblocked serial automatic feature extraction algorithm, and the right column indicates the three experimental evaluation metrics of the parallel SAE feature extraction algorithm.

We focused on the impact of the block-based parallel automatic extraction algorithm and unblocked serial automatic extraction algorithm on anomaly detection. We experimented from the following two aspects. (1) The effect of different feature extraction methods on anomaly detection: because we did not know whether the extracted shallow characteristics or deeper characteristics have a greater impact on the experimental results, we extracted the features to be compared from the three hidden layers of the stacked automatic encoder for final anomaly detection. (2) Feature extraction efficiency comparison: the feature extraction efficiency comparison was on the time for feature extraction consumed by the training set, the test set, and the entire dataset. To ensure the fairness of the experiment, we did not adopt the feature selection algorithm to avoid differences in detection performance due to the different dimensions of features after feature selection.  Figure 4 shows the experimental evaluation metrics for nine machine learning classifiers of the unblocked serial automatic feature extraction algorithm and parallel automatic feature extraction method. The used nine machine learning classifiers include logistic regression, decision tree classifier, random forest classifiers, Naive Bayes, AdaBoost classifiers, SVM (linear), SVM (RBF), gradient boosting, and XGBoost classifiers.

From Figure 4, we can see that the features output by the final hidden layer can substantially improve the classification accuracy of each machine learning classifier, and anomaly detection classifiers other than the Naive Bayes classifier showed very good performance. The obtained three metrics of accuracy, recall, and *F*1_score clearly show that the traffic-blocked parallel automatic feature extraction algorithm is better than the unblocked serial feature extraction algorithm, which can effectively solve the problem of encryption traffic anomaly detection.

For feature extraction efficiency, we comprehensively compared the feature extraction time of the two feature extraction algorithms on the training set, test set, and the whole dataset. Since the time consumed by blocked automatic feature extraction algorithms in feature extraction varies, for better comparison, we use the average consumption time for the blocked parallel SAE feature extraction algorithm.  Table 2 compares the efficiency of the two feature extraction algorithms for extracting the encryption traffic feature.

The experimental results show that the parallel SAE automatic feature extraction algorithm adopted by the core of the DETD framework is superior to the unblocked serial automatic feature extraction algorithm in anomaly detection implementation, and the parallel automatic feature extraction algorithm in feature extraction consumed only 1/3 the time of the unblocked feature extraction algorithm, which can greatly reduce the anomaly detection delay caused by feature extraction.

### 5.2. Why Did We Choose the L1 Regularization Feature Selection Algorithm?

Feature selection can effectively reduce computational cost and largely avoid classification accuracy degradation due to abnormal factors such as noise. In this section, we used a stacked self-encoder to extract encryption traffic features. The above experimental results show that the third hidden layer output has the best characteristics. To comprehensively compare the advantages of the L1 regularization-based feature selection algorithm over other selection algorithms, we chose the variance threshold method (VT), chi-square test, cross-validation recursive feature elimination, decision tree, and feature selection algorithm commonly used in random forest species. The experiment was performed in the following two aspects: (1) the feature set dimension after feature selection; and (2) the feature set performance on the weak classifier after feature selection. From the above experiment, we selected the Naive Bayes classifier as the weak classifier.

**Figure 5:**
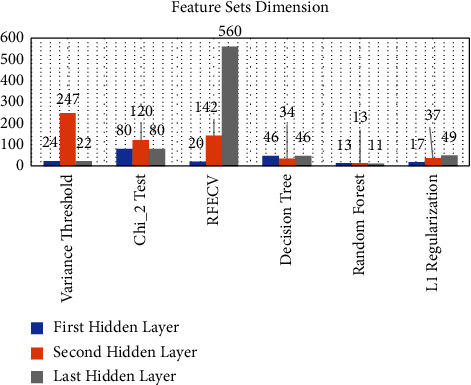
Dimensions of feature sets filtered by six feature selection algorithms.

Figure 5 shows the size of the feature set filtered by the six feature selection algorithms.  Table 3 shows the performance of the feature set filtered by the six feature selection algorithms on Naive Bayes.

As seen from Figure 5 and Table 3, the feature extraction algorithm based on L1 regularization, decision tree, random forest, and the feature selection algorithm is more advantageous when considering computational cost and time consumption. The feature set dimension does not exceed 50. The computational complexity is not high, and the result of the weak classifier Naive Bayes is satisfactory.

Moreover, the feature set obtained by random forest and L1 regularization screening is 99.559% and 99.662%, respectively, when passing through the Naive Bayes classifier. Therefore, in the subsequent experiments, we compared random forest and L1 regularization as feature selection algorithms.

### 5.3. AdaBoost Classifiers vs. Other Machine Learning Classifiers

**Figure 6:**
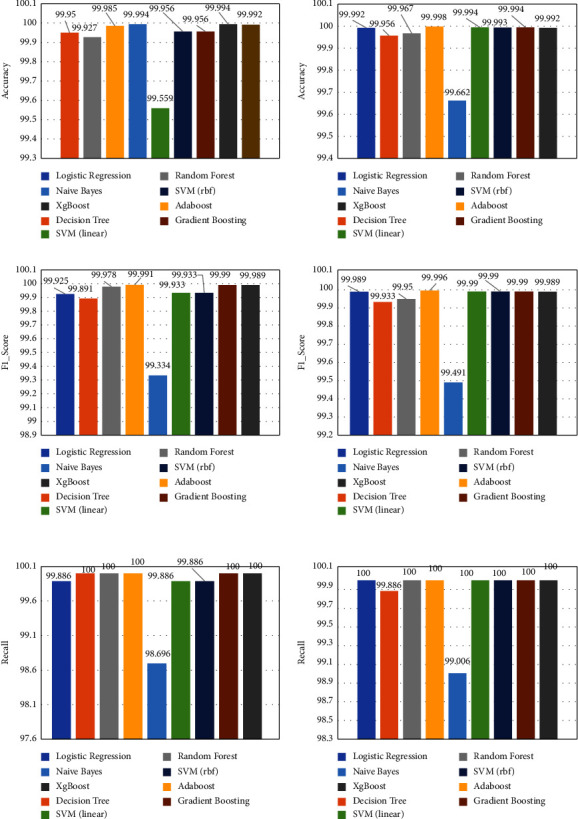
The left column indicates the three experimental evaluation metrics (accuracy, *F*1_score, and recall) of random forest selection algorithms for machine learning classifier, and the right column indicates the three experimental evaluation metrics of L1 regularization-based algorithms for classifier.

In this part, we verified the anomaly detection performance of AdaBoost and other anomaly detection classifiers after random forest and L1 regularization feature selection. To comprehensively compare the advantages of the AdaBoost classifier, we have slected a total of nine commonly used machine learning classifiers: logistic regression, decision tree classifier, random forest classifiers, Naive Bayes, AdaBoost classifiers, SVM (linear), SVM (RBF) gradient boosting, and XGBoost classifiers, for a total of nine commonly used machine learning classifiers.  Figure 6 shows the three test evaluation metrics for nine classifiers of two feature selection methods (serial automatic feature extraction algorithm and parallel automatic feature extraction method).

The above three experimental evaluation metrics show that the feature set after screening by the two feature selection algorithms has excellent performance on various anomaly detection classifiers, especially the AdaBoost anomaly detection classifier. The accuracy of the feature set based on the L1 feature selection algorithm on the AdaBoost classifier is as high as 99.998%, which is higher than that of the random forest feature selection algorithm.

## 6. Conclusions

Identifying malicious traffic without decryption is currently a major challenge in anomaly detection problems. However, the existing methods always require tedious analysis of various traffic features and attack features to extract features. Aiming at this deficiency, we propose a DETD anomaly detection framework, which is applied to the field of encryption traffic anomaly detection based on deep feature automatic feature extraction. The experimental results show that the proposed DETD framework has a huge advantage in extracting encrypted traffic features. The anomaly detection accuracy of DETD is as high as 99.998%, which outperforms other encryption traffic detection algorithms, such as the autoencoder-based method and the CNN-based approach. In other words, these results show that DETD is better than the recently proposed deep learning anomaly detection frameworks, resulting in our proposed framework being suitable for encrypted traffic intrusion detection. In future work, we will further investigate the classification problem of weakly labeled samples and unlabeled samples based on anomaly detection. Moreover, how to design various corresponding solutions for different types of encrypted traffic anomalies is another research direction.

## Figures and Tables

**Algorithm 1:**
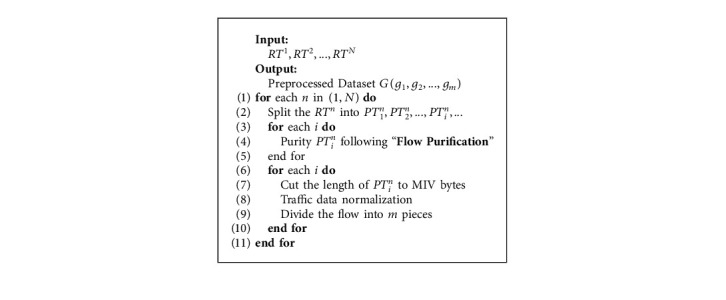
Preprocessing algorithm.

**Algorithm 2:**
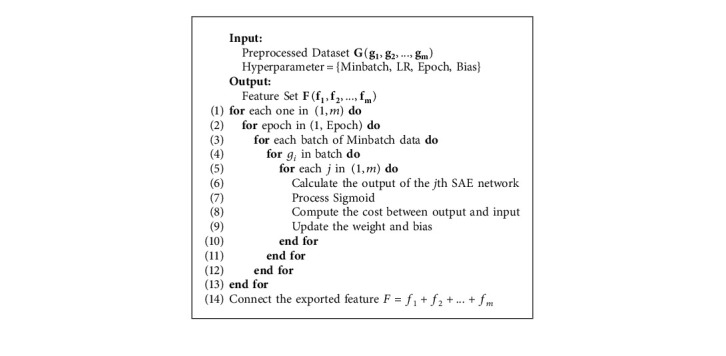
Parallel automatic feature extraction algorithm.

**Table 1:** Accuracy comparison of DETD and eight current encryption traffic intrusion detection algorithms.

Algorithms	Description	Accuracy (%)
Yang et al. [17]	Autoencoder	96.190
	CNN	97.901

Zeng et al. [18]	1D-CNN + L1 regularization	99.851
	LSTM + L1 regularization	99.222
	SAE + L1 regularization	98.741

Anderson and Mcgrew [10]	SPLT + BD + TLS + HTTP + DNS	99.993
	SPLT + BD + TLS + HTTP	99.983
	SPLT + BD + TLS + DNS	99.988
	HTTP + DNS	99.985

Anderson and Mcgrew [11]	Meta + SPLT + BD + TLS + SS	99.601
	Meta + SPLT + BD + TLS	99.610
	TLS	98.202
	Meta + SPLT + BD	98.900

Anderson et al. [12]	Linear regression	99.281
	L1-logistic regression	98.972
	Random forest	99.990
	MLP	99.542

Odiathevar et al. [30]	AE + MW + IGMM	97.435

Kim et al. [31]	RVAE	97.534

Wang et al. [32]	Model collaboration	94.353

Our proposed DETD	PASE + RF + adaboost/gradient boosting	99.994
	PASE + L1_R + adaboosting	**99.998**

The bolded values represent the best-performing result.

**Table 2:** Comparison of feature extraction efficiency of two automatic feature selection algorithms.

Amount of data	Time consumed by serial feature extraction (s)	Time consumed by parallel feature extraction (s)
360000	3984.83	1382.54
800000	8847.78	3070.24
1160000	12835.74	4453.67
Enhanced effect	When using the parallel blocked feature extraction algorithm: the time used by the parallel feature extraction characteristic is about 34% of that of the time consumed by the unblocked algorithm, and it greatly improves the feature extraction efficiency	

**Table 3:** Performance of feature set filtered by six feature selection algorithms on Naive Bayes.

FS	Accuracy (%)	*F*1_score (%)	Recall (%)	AUC_score (%)
VT	79.050	73.333	86.347	80.872
Chi_2 test	76.329	68.446	76.959	76.487
RECFV	98.392	97.581	97.528	98.175
Decision tree	98.581	97.827	95.765	97.878
Random forest	99.559	99.335	98.697	99.344
L1_R	**99.662**	**99.491**	**99.006**	**99.498**

The bolded values represent the best-performing result.

## Data Availability

The datasets used in this paper are open, which can be downloaded from the Internet.
